# Structure characterization and antioxidant activity analysis of polysaccharides from Lanzhou Lily

**DOI:** 10.3389/fnut.2022.976607

**Published:** 2022-08-05

**Authors:** Dandan Gao, Hong Chen, Honghai Liu, Xuhua Yang, Penghui Guo, Xin Cao, Yong Cai, Hongwei Xu, Jutian Yang

**Affiliations:** ^1^College of Life Sciences and Engineering, Northwest Minzu University, Lanzhou, China; ^2^Technology Research and Development Center, Gansu Tobacco Industry Co. Ltd, Lanzhou, China

**Keywords:** Lanzhou Lily, polysaccharide, extraction, structural characteristic, antioxidant activity

## Abstract

Lanzhou Lily (*Lilium davidii var. unicolor*) is a traditional medicinal plant and popular edible vegetable bulb in China. In this study, the polysaccharides of Lanzhou Lily (LLPs) were extracted by polyethylene glycol-based ultrasonic-assisted enzymatic extraction method (PEG-UAEE). The optimum process conditions were obtained by single-factor experiments and response surface methodology (RSM). Then, the preliminarily structure of LLPs was characterized by HPLC, FT-IR, and SEM, and its antioxidant activities were evaluated. The results showed that LLPs yield reached 14.75% under the optimized conditions: E/S ratio 1,400 U/g; pH 5.0, ultrasonic time 30 min; and ultrasonic temperature 50 °C. The LLPs has pyranoid ring, uronic acid, and the characteristic absorption peaks of -OH, C = O, and C-H. The results of scanning electron microscope indicated that the LLPs had irregular distribution, dispersed structure, and many holes. The HPLC analysis showed that the LLPs were heteropolysaccharide containing galactose (6.36%), glucose (76.03%), rhamnose (2.02%), and arabinose (7.09%). Moreover, the LLPs showed obvious antioxidant effect *in vitro*.

## Introduction

Lanzhou Lily is a plant of morningstar lily bulb, called “sweet lily.” It is the only sweet and edible lily in China with white color and sweet taste (1). Modern researches have shown that Lanzhou Lily has high edible, medicinal and ornamental value, and has further development and application potential (2). Lanzhou Lily contains massive pectin, alkaline elements and natural phospholipids, as well as abundant plant protein and cellulose. Lanzhou Lily has the functions of strengthening the spleen, nourishing the stomach, delaying aging and preventing gout. Lanzhou Lily also has many biological activities, including antioxidant, antitumor, hypoglycemic, immunomodulatory, and other pharmacological effects (3, 4), thus protecting human spleen, lung, and other organs, is beneficial to human body.

Polysaccharide is a natural high polymer linked by aldose or ketose through glycosidic bonds, and widely exists in plants. It is an important macromolecular substance in organisms and a basic substance maintaining normal life activities. Polysaccharides are mostly polymerized by several monosaccharides in a certain proportion. Polysaccharides vary in the molecular composition and weight (5). The content of polysaccharides in Lanzhou Lily is about 14.58 mg/g, which exert pharmacological effects such as antioxidant, hypoglycemic, anti-tumor, anti-fatigue and immune regulation (6). The principle of extracting Lanzhou Lily polysaccharide is to obtain the maximum extraction rate and reserve the active structure. It is of great significance to select an appropriate extraction method for polysaccharides.

The commonly used extraction methods of lily polysaccharide include hot water extraction, ultrasonic extraction, microwave extraction, and enzymatic extraction (7). Hot water extraction method is easy to operate and realize, but the cost is high and the yield is low. Ultrasonic extraction method can dramatically shorten the extraction time and improve the extraction efficiency, but it has high requirements on the container and large noise. Microwave extraction has the advantages of short time and simple equipment, but it is easy to be affected by external factors. Enzymatic extraction reaction conditions mild, less side reaction, but it needs a long time.

PEG can be used as solvent and cosolvent due to the good water solubility and low relative molecular weight (8). In this work, we developed an ultrasonic assisted enzymatic extraction method to obtain LLPs using PEG as the solvent. The method has developed a kind of efficient extraction technique to extract polysaccharides from various biomaterials. As expected, compared to ultrasonic assisted, PEG reagent provides -OH groups to enhance the interaction of polysaccharides which could improve polysaccharides yield. The optimal extraction process of LLPs was obtained by single-factor experiments and RSM with box-behnken design. Then, FT-IR, SEM, and HPLC were used to analyze the compositions and structural characteristic of LLPs. Moreover, antioxidant activities of LLPs were also evaluated.

## Materials and methods

### Materials and reagents

Fresh Lanzhou Lily at the mature stage of commercial was purchased from the local markets at Lanzhou city (Gansu, China). Cellulose and pectinase were obtained from Solarbio Biological Reagent Co., Ltd. (Beijing, China). 1,1-diphenyl-2-trinitrophenylhydrazine (DPPH), Trifluoroacetic acid (TFA) and monosaccharide standard products were from Sigma-Aldrich Chemical Co., Ltd. (Louis, USA). PEG and other reagents were all analytically pure and purchased from Sinopharm Chemical Reagent Co., Ltd. (Beijing, China).

### Extraction of LLPs

Fresh Lanzhou Lily was cleaned, and then was frozen-dried by a vacuum freeze dryer (LGJ-100F, Thermo Co., USA). The dried lily powder was crushed and collected through an 80 mesh sieve. Then the power was degreased twice with n-hexane (M: V = 1:3) for 6 h every time, dried and collected for further use. Three gram defatted lily powder was accurately weighed, 45 mL 30% PEG-400 solution was added at the ratio of material to liquid of 1:15, and a mixed enzyme (cellulose: pectase = 1:2) was added. After pH adjustment, ultrasonic cleaning machine (SB-500DTY, Ningbo Xinzhi Biotechnology Co., China) was used for extracting under a ultrasonic power of 250 W. Repeated the above process 3 times. The extracts were mixed, centrifuged at 5,000 rpm for 10 min (Heraeus Multifuge X1R, Thermo, America), and the supernatant was taken. Sevage reagent (n-butanol: chloroform = 1: 5, v/v) was added to the supernatant to remove proteins. The solution was centrifuged (5,000 r/min, 10 min) and the upper layer solution was collected. Then 3 times (v/v) anhydrous ethyl alcohol was added for alcohol precipitation, and precipitate was formed after standing at 4°C for 24 h. The precipitate was recovered by centrifugation at 5,000 r/min for 10 min, and was successively washed by ethanol, petroleum ether and diethyl ether. The precipitate was dried in a DHG-9030A oven (Shanghai Grows Instrument Co., Ltd., China) to obtain LLPs. The extraction rate (%) of LLPs was calculated as follows:


(1) $$\begin{array}{r} R\;=\;\frac{m_{1}}{m_{0}}\times 100 \end{array}$$

Where R is the extraction rate of LLPs (%); m_1_ is the weight of lily polysaccharide extracted (g); m_0_: the weight of the extracted lily powder (g).

### Experimental design of optimization of extraction conditions

The effects of E/S ratio (700, 1,400, 2,100, 2,800, and 3,500 U/g), pH (3.0, 4.0, 5.0, 6.0, and 7.0), ultrasound time (10, 20, 30, 40, and 50 min), and ultrasound temperature (30, 40, 50, 60, and 70°C), on the extraction rate of LLPs were investigated. The experiments were conducted in triplicate.

According to the results of the single-factor experiments, X_1_ (E/S ratio), X_2_ (pH value), X_3_ (ultrasound time), X_4_ (ultrasound temperature) were selected as the independent variable, and Y (crude polysaccharide extraction rate) was used as the response value. A four-factor three-level Box-Behnken response surface experimental design (BBD) was carried out. Three levels (−1, 0, and 1) were designed for each independent variable, the influencing factors and level design were shown in Table 1. Each experiment was repeated 3 times. By analyzing the results, a linear quadratic model was obtained as follow:


(2) $$\begin{array}{r} Y\;=\;B_{0}\;+\;\overset{k\;=\;3}{\underset{i\;=\;1}{\sum }}B_{i}X_{i}\;+\;\overset{k\;=\;3}{\underset{i\;=\;1}{\sum }}B_{ii}X_{i}^{2}+\overset{k\;=\;3}{\underset{i\;=\;1}{\sum }}B_{ii}X_{i}Y_{i} \end{array}$$

Where Y is the response variable (LLPs extraction yield, %); B_0_, B_i_, B_ii_, and B_ij_ are the regression coefficients of variables for the intercept, linear, quadratic, and interaction terms, respectively; X_i_ and X_j_ are the independent variables (i ≠ j).

**Table 1:** The process parameters setting for LLPs extraction, according to Box-Benkhen design.

**Factor**	**Level**		
	**−1**	**0**	**1**
X_1_-E/S ratio /(U/g)	700	1,400	2,100
X_2_-Extraction pH value	4.0	5.0	6.0
X_3_-ultrasound times/min	20	30	40
X_4_-ultrasonic temperature/ °C	40	50	60

### FT-IR spectrometric analysis of LLPs

A sample pellet was prepared by mixing 1 mg LLPs with 500 mg KBr, mortared, and pressed 5 min by a HYP-15 machine (Tianjin Port East Technology Co., Ltd, China). The sample was scanned for 20 min between 4,000 and 400 cm^−1^ using a Fourier infrared spectrometer (FTIR-650, Tianjin Port East Technology Co., Ltd, China) to analyze the chemical bonds and functional groups of LLPs (9).

### SEM analysis of LLPs

LLPs were fixed on the sample table with conductive adhesive, and the samples coated with gold sputtering were scanned at 20 kV with Zeiss tungsten filament scanning electron microscope (ZEISS EVO18, Carl Zeiss AG, Bruker Co., Germany) to observe the sample morphology under different multiples (10).

### Monosaccharide composition analysis

The monosaccharide components of LLPs were determined by HPLC (Agilent1260, USA) coupled with 3-methyl-1-phenyl-2-pyrazolin-5-one (PMP) pre-column derivatization (4.6 × 250 mm, 5 μm, Agilent Co., USA) (9, 11). 10.00 mg crude LLPs sample was precisely weighed and 5 mL of 2 M trifluoroacetic acid (TFA) solution was added. Then the sample was hydrolyzed in water bath at 100°C for 5 h. After cooling, the pH was adjusted to 7.0 using 3 M NaOH, then centrifuged at 5,000 r/min for 10 min. 0.2 mL of 0.5 M PMP/methanol solution and 0.3 M NaOH were added into 0.2 mL polysaccharide hydrolysate. After swirling mixing, water bath at 70°C for 1 h was performed. Then, 1 mL chloroform and 0.2 mL HCL (0.3 M) were mixed, centrifuged to get the supernatant and filtered through 0.22 μm membrane for monosaccharide composition analysis. Monosaccharide standards (rhamnose, glucose, galactose, fructose, and arabinose) were treated as same as described above.

HPLC analysis was performed using an Agilent 1260 HPLC system with a diode array UV-Vis detector (DVD), and an Agilent ZORBAX Eclipse XDB-C18 analytical HPLC column (4.6 × 250 mm, 5 μm). The mobile phase consisted of 20.0 mM phosphate buffer (pH 6.8) (A) and acetonitrile (B) in a ratio of 81: 19 (v/v). The column temperature was 28°C and the flow rate was 1 mL/min. The injection volume was 5 μL and the detection wavelength was 250 nm.

### *In vitro* antioxidant activity assay

#### ·OH radical scavenging assay

The ·OH radical scavenging activity of LLPs were determined by the method of Zhou et al. (12). The solutions of LLPs were prepared at concentrations of 0.0, 0.2, 0.4, 0.6, 0.8, and 1.0 mg/mL, respectively. An aliquot of 1.0 mL sample solution, 1.0 mL o-phenanthroline ethanol solution (0.75 mM) and 1.0 mL FeSO_4_ (0.75 mM) were mixed, and placed in a water bath at 37°C for 30 min. After that, 1.0 mL H_2_O_2_ (0.01%) and 2.0 mL PBS (pH 7.4) were added to the mixture. When the mixture was evenly mixed, incubated it in a water bath at 37°C for 15 min. After cooling, the absorbance value A_x_ of the sample solution was determined at 510 nm, and distilled water was used as blank control. V_C_ (0.0, 0.2, 0.4, 0.6, 0.8, and 1.0 mg/mL) was used as positive control. The ·OH radical scavenging activity was calculated by the following formula:


(3) $$\begin{array}{r} E\;=\;\left(1-\frac{A_{x}-A_{xo}}{A_{o}}\right)\times 100 \end{array}$$

Where E is ·OH radical scavenging activity (%); A_o_ is the absorbance value of blank control; A_x_ is the absorbance value of the samples; A_xo_ is the absorbance value of the sample without ·OH radical.

#### DPPH radical scavenging assay

The DPPH radical scavenging activity of LLPs was measured by the method of Liu et al. with slight modification (13). 2 mL LLPs samples at different concentrations (0.0, 0.2, 0.4, 0.6, 0.8, 1.0 mg/mL) were mixed with 2 mL DPPH solution (0.2 mM), reacted at room temperature for 30 min in a dark environment. And the absorbance value of the mixed solution was measured at 517 nm. The control 1 was composed by 2.0 mL DPPH and 2.0 mL distilled water, the control 2 was composed by 2.0 mL sample solution and 2.0 mL distilled water, and V_C_ group was used as positive control. The DPPH radical scavenging ability was calculated according to the following equation (14):


(4) $$\begin{array}{r} E=\left(1-\frac{A_{a}-A_{c}}{A_{b}}\right)\times 100 \end{array}$$

Where A_a_ is the absorbance of the samples; A_b_ is the absorbance of the control 1; A_c_ is the absorbance of the control 2.

#### ·${\text{O}}_{2}^{-}$ radical scavenging assay

The ·${\text{O}}_{2}^{-}$ radical scavenging activity of LLPs was determined according to the method described previously (15, 16). The LLPs samples and V_C_ were formulated into solutions with concentration gradients of 0.0, 0.2, 0.4, 0.6, 0.8, and 1.0 mg/mL, respectively. After adding 1.0 mL LLPs solution and 4.5 mL Tris-HCl buffer (pH 8.2), the mixture was incubated at 25°C water bath for 10 min. Then 0.4 mL of 25 mM catechol solution was added and fully mixed, and incubated at 25°C water bath for 5 min. Finally, 1 mL of 8 M hydrochloric acid was added to terminate the reaction, and the absorbance values were measured at 320 nm. Distilled water instead of polysaccharide solution was used as blank control and V_C_ group as positive control. The scavenging activity was calculated as follows:


(5) $$\begin{array}{r} E=\frac{A_{ii}\;-\;A_{i}}{A_{ii}}\times 100 \end{array}$$

Where A_i_ is the absorbance of the control; A_ii_ is the absorbance of the samples.

### Statistical analysis

SPSS 25.0 software was used to analyze the data, and the results were expressed as mean ± standard deviation. An ANOVA analysis was performed. The design expert software (Version 8.0.6, Stat-Ease, Inc., Minneapolis, MN, USA) was used for the experiment design. All the experiments or analyses were carried out in triplicate. The 50% inhibitory concentrations (IC_50_ values) were calculated by Probit analysis method using the SPSS software.

## Results and discussion

### Effects of E/S ratio, pH, ultrasonic time, and ultrasonic temperature on the extraction yield of LLPs

**Figure 1:**
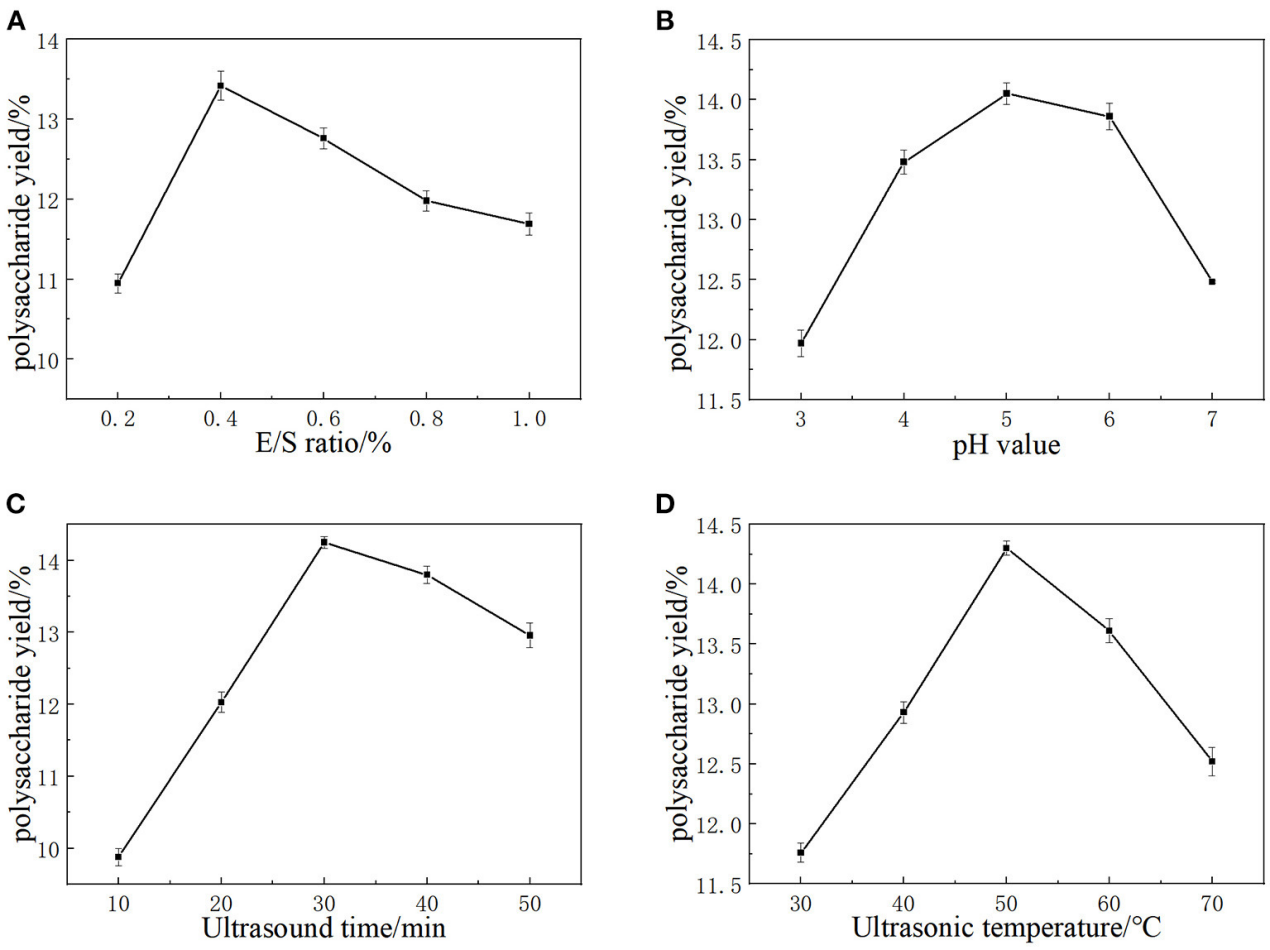
The effects of E/S ratio **(A)**, extraction pH **(B)**, Ultrasound time **(C)**, and Ultrasonic temperature **(D)** on the yield of LLPs.

Cellulase and pectinase are normally able to degrade cellulose and pectin in plant cell walls, which could improve the extraction yield of LLPs (17). The cellulase and pectinase amounts can markedly affect the extraction yield of the LLPs. As a consequence, E/S ratio (700, 1,400, 2,100, 2,800, 3,500 U/g) on the extraction yield were investigated with enzymolysis pH of 5.0, ultrasonic time of 30 min, and ultrasonic temperature of 50°C. As shown in Figure 1A, the extraction ratio of LLPs increased from 10.95 to 13.42%, and then fall solely as E/S ratio increased from 1,400 to 3,500 U/g. The possible reason for this is the increase of cellulase and pectinase can effectively destroy the lily cells, resulting in more polysaccharide overflow and higher polysaccharide yield. However, when the E/S ratio was large, the polysaccharide glycosidic bond was partially hydrolyzed due to substrate saturation, and the extraction decreases (18). Considering the cost, E/S ratio of 1,400 U/g was selected as the center point of RSM.

It has been shown that the same type of enzymes from different origins have different activities and different pH optimums. PH value will affect the degree of dissociation of the essential groups on the enzyme activity center. The enzyme shows different activities under different pH conditions, and too high or too low pH value will reduce the enzymatic hydrolysis efficiency. Hence, pH values (3.0, 4.0, 5.0, 6.0, and 7.0) were selected as a univariate variable. As Figure 1B showed that the extraction ratio of LLPs increased with pH from 3.0 to 5.0, reaching a maximum value of 14.05 ± 0.09% at pH 5.0, and then decreased continuously with pH from 5.0 to 7.0 (17). The results could be that cellulase enzyme achieved only limited solubilization (19). Based on our results, a pH value of 5.0 was chosen as the RSM central point.

Ultrasound treatment could improve the efficiency of LLPs extraction, because of high pressure, temperature and shear force generated by the ultrasonic wave may break chemical bonds of polysaccharide in cell walls (20). The increase of ultrasonic time provides the conditions for ultrasonic wave to destroy more lily cells, so that more polysaccharides can be dissolved in PEG solution. As a result, the effect of ultrasonic time (10, 20, 30, 40, 50 min) on the extraction yield were investigated when the others extraction parameters were set as follows: E/S ratio 5.0, pH 5.0, and ultrasonic temperature 50°C. Figure 1C shows that polysaccharide yield reached the maximum value of 14.25 ± 0.04% when ultrasonic time was 30 min. If the ultrasonic time exceeded 30 min, the LLPs extraction rate decreased significantly. When the ultrasonic time was <30 min, more polysaccharides had enough time to dissolve in the container with the extension of time, so the yield of polysaccharides increased. When the ultrasonic time exceeded 30 min, the high pressure, temperature and shear force generated by ultrasonic may destroy the chemical bonds of polysaccharides, leading to the hydrolysis of some polysaccharides, and the extraction rate of LLPs decreased significantly. Cai et al. (21) reported that with extended ultrasonic times, strong mechanical shock damages the dissolved ingredients and leads to the hydrolysis of some polysaccharides, which is consistent with our findings. Hence, 30 min was chosen as the optimal ultrasonic time and the RSM central point.

Temperature is also an important factor in the extraction of polysaccharides. The diffusion coefficient and solubility of polysaccharide in the extracting solvent was enhanced at a higher temperature, is possibly caused by the increase of the extraction rate of polysaccharides (22). Therefore, ultrasonic temperature (30, 40, 50, 60, 70°C) was studied under the conditions of fixed E/S ratio 5.0, pH 5.0, ultrasonic time 30 min. According to Figure 1D, the yield of LLPs increased with temperature in the range of 30–50°C, and reached the maximum value of 14.30 ± 0.02% at 50°C. After that, the yield of LLPs decreased with the increase of temperature (50–70°C). The main reason for this phenomenon was that the solubility and diffusion coefficient of polysaccharides in the extraction solvent increase at higher temperature, so that the amount of polysaccharides dissolved in the solution increases. When the temperature was too high, the biological potential of the enzyme was destroyed and lost activity, and irreversible changes occurred. Meanwhile, the polysaccharide structure was damaged and degraded in the high temperature environment, so the extraction rate of polysaccharide decreased. Cai et al. (21) reported that the mild conditions are beneficial to maintain the biological potential of enzyme, while a higher temperature will destroy the biological potential of enzyme (23). In conclusion, the optimal ultrasonic temperature was 50°C, which was used as the center point of RSM.

### Analysis of the response surface

#### Statistical analysis and the model fitting

According to the principle of BBD central combined test, 29 groups were designed by Design-expert 8.0.6 software. The experimental Design level and experimental results of independent variables were summarized in Table 2. The extraction rate varied from 9.28 to 14.75%, with the maximum value being obtained under the conditions of E/S ratio 1,400 U/g, pH 5.0, ultrasonic time 30 min, and ultrasonic temperature 50°C. The second-order polynomial equation of the influence of the three test variables on the response variables was obtained as follows:


(6) $$\begin{array}{r} Y\;=\;14.39-0.13X_{1}\;+\;0.52X_{2}-0.28X_{3}\;+\\ 0.22X_{4}-0.56X_{1}X_{2}\;+\;1.50X_{1}X_{3}+0.43X_{1}X_{4}+\\ 0.47X_{2}X_{3}-0.26X_{2}X_{4}\;+\;0.017X_{3}X_{4}-1.12X_{1}^{2}\\ -1.43X_{2}^{2}-2.17X_{3}^{2}-2.04X_{4}^{2} \end{array}$$

Where Y is the LLPs yield (%); X_1_, X_2_, X_3_, and X_4_ are the coded values of the tested E/S ratio (%), extraction pH value, ultrasonic times (min), and ultrasonic temperature (°C), respectively.

**Table 2:** Box-Benkhen design of the independent variables and experimental values of LLPs yield (Y).

**No**.	**Factor**				**Y (LLPs yield)/%**
	**X_**1**_-E/S ratio/(U/g)**	**X_**2**_-pH value**	**X_**3**_-ultrasonic times/min**	**X_**4**_-ultrasonic temperature)/**°**C**	
1	700	4.00	30.00	50.00	11.05
2	2,100	4.00	30.00	50.00	11.88
3	700	6.00	30.00	50.00	12.91
4	2,100	6.00	30.00	50.00	11.50
5	1,400	5.00	20.00	40.00	10.27
6	1,400	5.00	40.00	40.00	9.69
7	1,400	5.00	20.00	60.00	10.61
8	1,400	5.00	40.00	60.00	10.10
9	700	5.00	30.00	40.00	11.76
10	2,100	5.00	30.00	40.00	10.28
11	700	5.00	30.00	60.00	11.31
12	2,100	5.00	30.00	60.00	11.56
13	1,400	4.00	20.00	50.00	10.88
14	1,400	6.00	20.00	50.00	11.24
15	1,400	4.00	40.00	50.00	9.38
16	1,400	6.00	40.00	50.00	11.62
17	700	5.00	20.00	50.00	12.87
18	2,100	5.00	20.00	50.00	9.98
19	700	5.00	40.00	50.00	9.28
20	2,100	5.00	40.00	50.00	12.39
21	1,400	4.00	30.00	40.00	9.89
22	1,400	6.00	30.00	40.00	11.48
23	1,400	4.00	30.00	60.00	10.95
24	1,400	6.00	30.00	60.00	11.49
25	1,400	5.00	30.00	50.00	14.75
26	1,400	5.00	30.00	50.00	13.97
27	1,400	5.00	30.00	50.00	14.18
28	1,400	5.00	30.00	50.00	14.64
29	1,400	5.00	30.00	50.00	14.43

The results of analysis of variance (ANONA) analysis were shown in Table 3. The model had a high F value (81.95) and a low *P*-value (< 0.0001), indicating that the model was statistically significant and effective. Where *R*^2^ = 0.9879, indicating that 98.79% of the results can be explained by this model adjustment coefficient, and 1.21% of the results can't be explained. The $R_{\text{adj}}^{2}$ = 0.9759, closed to *R*^2^, indicating that the model has a good fit with the actual, the correlation between the observed value and the predicted value is good (13). The low coefficient of variation (C.V. = 2.14%) showed high accuracy of the model. The significance of each coefficient was listed in Table 3. The major factor (X_2_, X_3_, and X_4_), minor factor (${\text{X}}_{1}^{2}$, ${\text{X}}_{2}^{2}$, ${\text{X}}_{3}^{2}$, and ${\text{X}}_{4}^{2}$) and interaction item (X_1_X_2_, X_1_X_3_, X_1_X_4_, and X_2_X_3_) had significant effects on the yield of LLPs (*P* < 0.01). However, the interaction term (X_2_X_4_ and X_3_X_4_) of the main factor (X_1_) had not a significant influence (*P* > 0.05). It can be seen that the influencing factors of the four variables were in the order of X_2_>X_3_>X_4_>X_1_, and the pH value had the greatest influence on the extraction rate.

**Table 3:** Analysis of variance and results of regression equation.

**Source**	**Sum of square**	**Df**	**Mean square**	**F value**	***P*-value**
Model	70.39	14	5.03	81.95	<0.0001**
X_1_	0.21	1	0.21	3.43	0.0851
X_2_	3.21	1	3.21	52.38	<0.0001**
X_3_	0.96	1	0.96	15.61	0.0014**
X_4_	0.59	1	0.59	9.54	0.008**
X_1_X_2_	1.25	1	1.25	20.44	0.0005**
X_1_ X_3_	9	1	9	146.69	<0.0001**
X_1_ X_4_	0.75	1	0.75	12.2	0.0036**
X_2_ X_3_	0.88	1	0.88	14.4	0.002**
X_2_ X_4_	0.28	1	0.28	4.49	0.0524
X_3_ X_4_	0.001	1	0.001	0.02	0.8896
${\text{X}}_{1}^{2}$	8.08	1	8.08	131.71	<0.0001**
${\text{X}}_{2}^{2}$	13.24	1	13.24	215.79	<0.0001**
${\text{X}}_{3}^{2}$	30.65	1	30.65	499.51	<0.0001**
${\text{X}}_{4}^{2}$	26.96	1	26.96	439.39	<0.0001**
Model	0.86	14	0.061		
Lack of Fit	0.44	10	0.044	0.43	0.8729
Pure Error	0.41	4	0.1		
Cor total	71.25	28			
	*R*^2^ = 0.9879		C.V. = 2.14%	$R_{\text{Adj}}^{2}$ = 0.9759	

*represents significant difference (P <0.05), **represents extremely significant difference (P <0.01).

#### Response surface plot and contour plot analyses of the extracted LLPs

**Figure 2:**
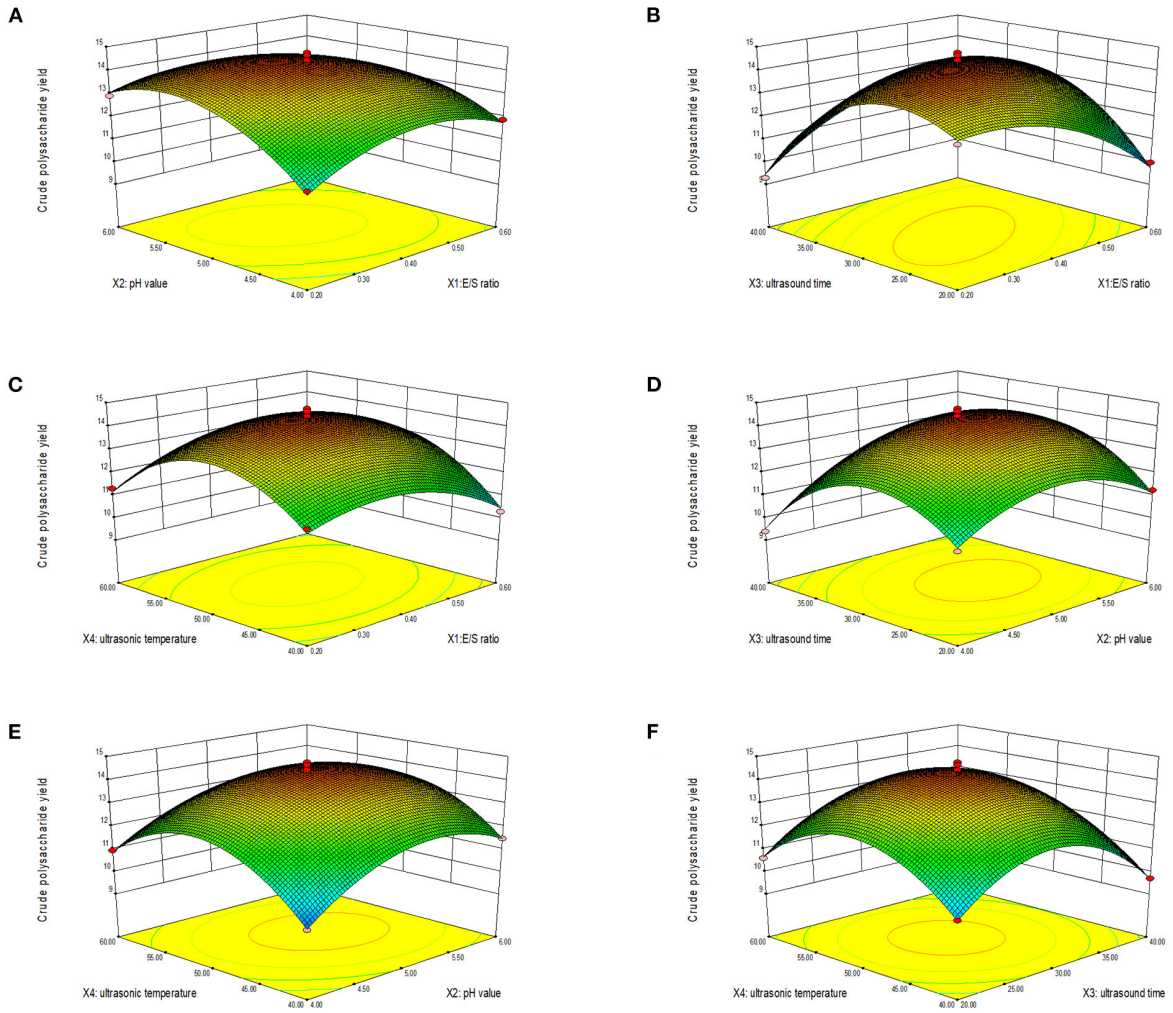
Response surface (3D) showing the effect of E/S ratio and pH value **(A)**, E/S ratio and ultrasonic time **(B)**, E/S ratio and ultrasonic temperature **(C)**, pH value and ultrasonic time **(D)**, pH value and ultrasonic temperature **(E)**, and ultrasonic time and ultrasonic temperature **(F)** on extraction yield of LLPs.

The three-dimensional response surface and contour diagram are shown in Figure 2. Each sub-figure shows the response surface and contour map of two factors to LLPs extraction rate, respectively. The influence of various factors on the response surface is staggered, and the degree of influence can be clearly reflected by the strength of the isoline in the contour map and the steep inclination of the response surface. Dense contour lines indicate steeper response surface and higher impact. The larger the distance between contour lines, the smaller the influence (24). Figures 2A–C showed that when pH value, ultrasonic time and ultrasonic temperature were fixed at the median value (level 0), the extraction rate reached the highest with the increase of E/S ratio. When the E/S ratio remained level 0, the extraction rate increased first with the increase of pH value, ultrasonic time and ultrasonic temperature, and then decreased. The results indicated that long ultrasonic time or high extraction temperature would increase the solubility rate of LLPs, but excessive ultrasonic time and extraction temperature would result in the hydrolysis of polysaccharides. A moderate high pH increased the activity of the enzyme, but a higher pH inhibited the activity of the enzyme. Figures 2D,E showed that when ultrasonic time and temperature were fixed at level 0, the highest extraction yield was observed with the increase of pH. The extraction rate increased first and then decreased with the increase of pH value, ultrasonic time and ultrasonic temperature. The interaction between ultrasonic time and ultrasonic temperature was shown in Figure 2F, when the ultrasonic time and temperature were fixed at level 0, the extraction rate was maximal. During the experiment, when E/S ratio, pH value, sonication time, and sonication temperature closed to 1,400 U/g, 5.00, 30 min, and 50°C, respectively, the polysaccharide extraction rate was maximized. The graphs of X_1_ and X_2_, X_1_ and X_3_, X_1_ and X_4_, X_2_ and X_3_ were steeper, and their contour lines can be seen to be ellipses. The graphs of X_2_ and X_4_, X_3_ and X_4_ were gentler, and their contour lines were circles. This result was consistent with the results of the ANOVE.

#### Validation of the predictive model

According to the mathematical prediction of the BBD, the optimal PEG-based UAEE conditions with the maximum LLPs yield (Y) obtained by the Design Expert software were: E/S ratio (X_1_) of 1,295 U/g, extraction pH value (X_2_) of 5.20, ultrasonic times (X_3_) of 28.95 min and ultrasonic temperature (X_4_) of 50.22°C. Under the optimal conditions, the extraction yield reached 14.75%, close to the predicted value of 14.39% by response surface. The ultrasound-assisted enzymatic extraction (25) showed a low extraction rate (9.62%) compared to our method, indicating the PEG-UAEE extraction of LLPs is effective.

### FT-IR spectrum of LLPs

**Figure 3:**
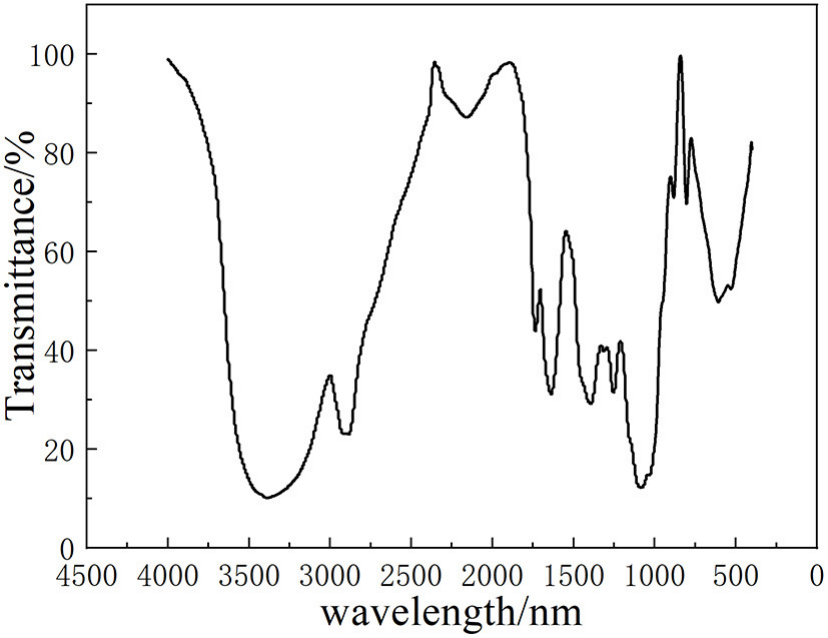
Infrared spectra of LLPs.

Figure 3 shows the Fourier infrared spectrum of LLPs. According to the spectrogram, the characteristic absorption peak at 3379.6 cm^−1^ was assigned to the O-H stretching vibration (26), the 2999.7 cm^−1^ peak was relegated to C-H stretching vibration. The rough absorption peak at 1540.5 cm^−1^ were caused by C=O stretching vibration of the carboxyl products in the polysaccharide, and suggested the existence of uronic acid (26). The broader band of LLPs at 1540.5 cm^−1^ indicated higher content of uronic acid. There was also an absorption peak at 1 080.6 cm^−1^, suggesting the LLPs has pyranose rings. The absorption peak at 773.5 cm^−1^ indicated that LLPs was α -D-glucan (25). This conclusion is generally consistent with Chen et al. (27) that the polysaccharide of tiger lily has the characteristics of pyranose.

### Microstructure of LLPs

**Figure 4:**
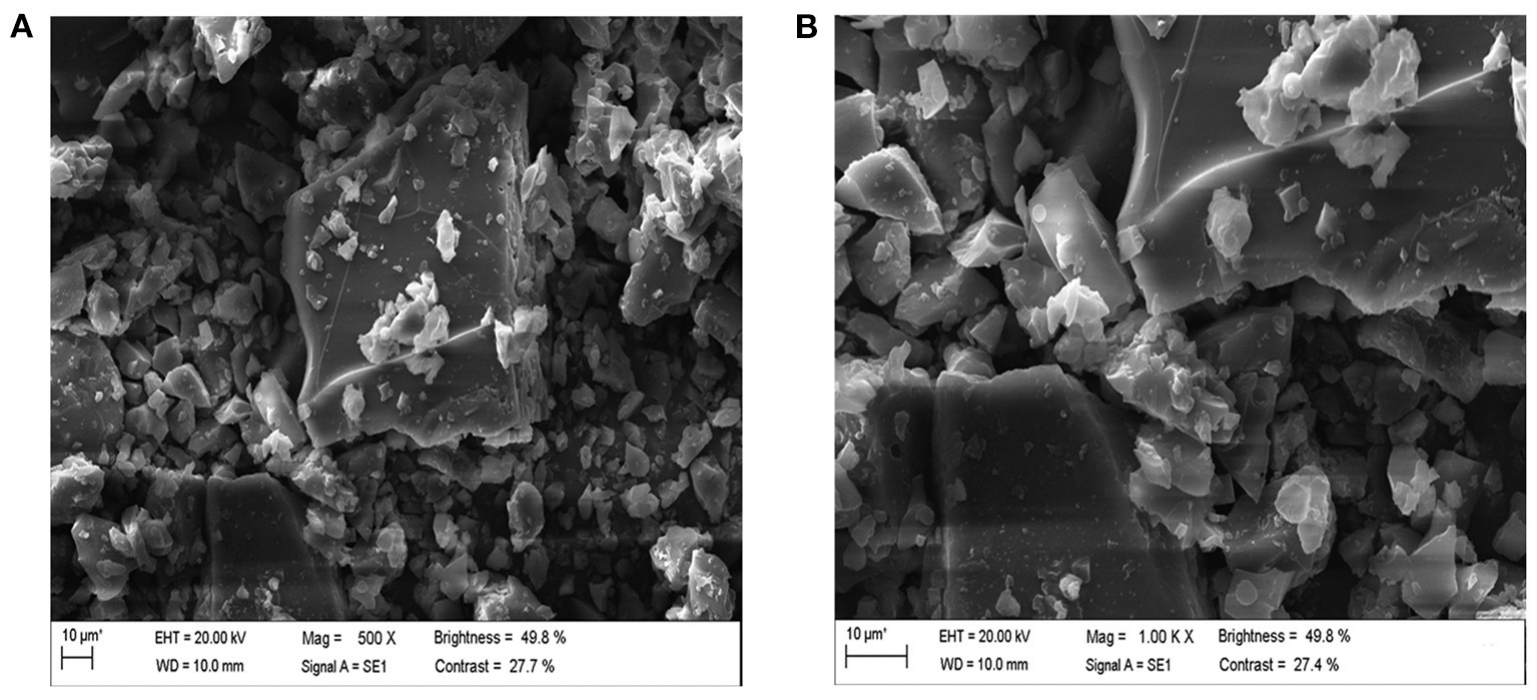
Scanning electron microscopy of LLPs [**(A)**: 500 X, **(B)**: 1.00 KX].

The data of SEM were presented in Figure 4. Sub-Figures 4A,B were 500X and 1000X images amplified under scanning electron microscopy. LLPs showed a fragmented and irregularly shaped morphology at magnifications of 500X (28). LLPs were composed of many small particles and clay-like clusters with irregular shapes and sizes. Most of the particles were massive, with irregular size and cracks (29, 30). The surface of LLPs when observed at magnifications of 1000X appeared to be relatively smooth, with some honeycombed cavities. LLPs presented irregular distribution, structure scattered, with many holes. Compared with the results of Hou et al. (31), the smooth surface of LLPs and the far difference in other characteristics may be due to the different treatment methods affecting the sample structure.

### Monosaccharide composition of LLPs

**Figure 5:**
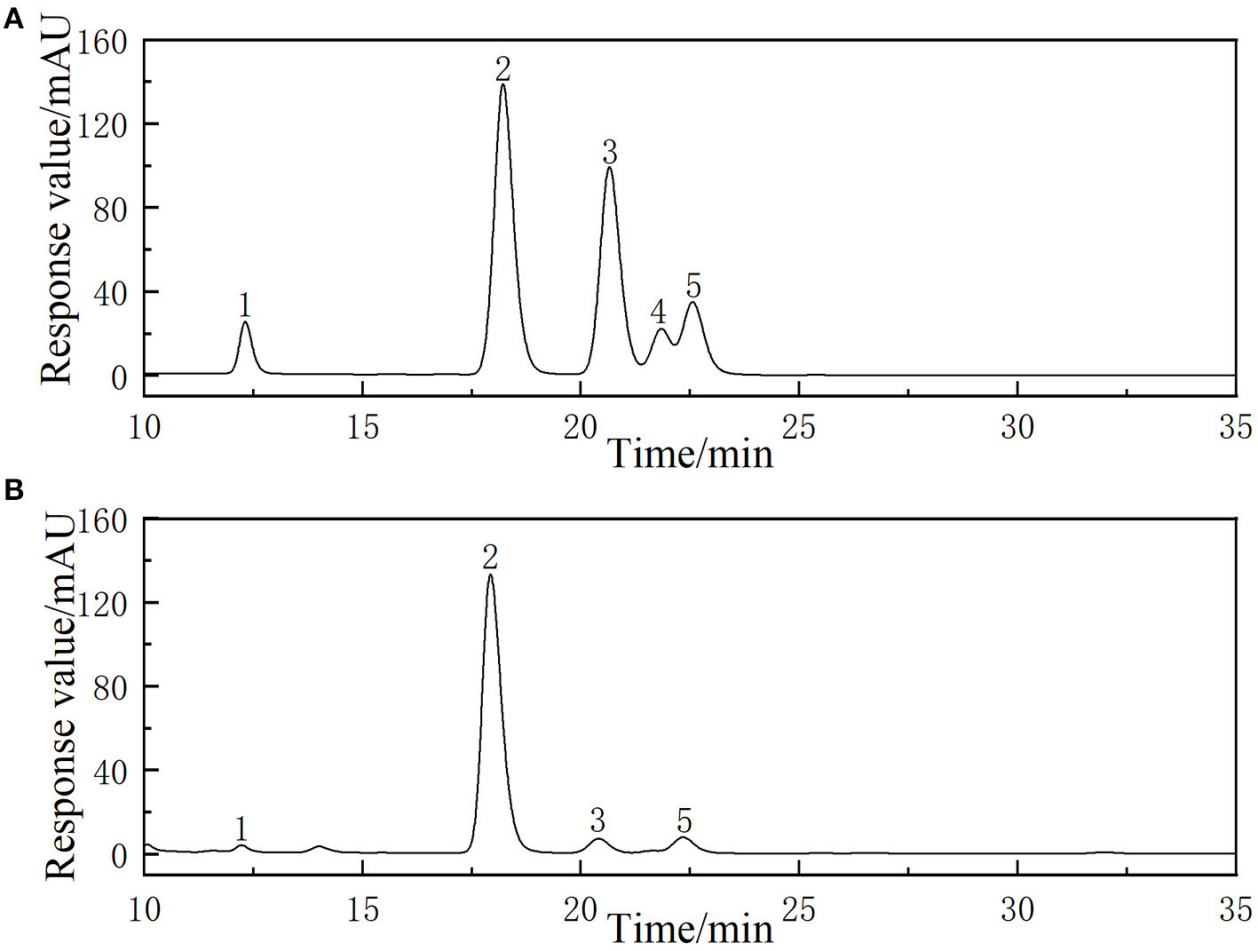
HPLC chromatogram of reference monosaccharides **(A)** and monosaccharides composition of LLPs **(B)**.

The chromatogram of standards (rhamnose, glucose, galactose, fructose, and arabinose) and the LLPs are shown in Figure 5. The results showed that the LLPs was mainly composed of galactose, glucose, rhamnose, and arabinose, with a molar ratio of 6.36: 76.03: 2.02: 7.09. Chen et al. (27) found that LLPs-3, a purified component of lily water-soluble polysaccharide, was mainly composed of arabinose, galactose, glucose and mannose, with a molar ratio of 2:2:2:1. Gao et al. inferred that l-rhamnopyranose, d-arabinofuranose, d-glucopyranose, and d-galactopyranose in the molar ratio of 1.88:2.13:1.00:2.50 were main monosaccharide type of a novel polysaccharide fraction (LP2-1) from the edible bulbs of Lilium lancifolium Thunb (32). The two results indicated that the main monosaccharide compositions of LLPs were similar.

### Antioxidant activity analysis

#### Scavenging hydroxyl radical

**Figure 6:**
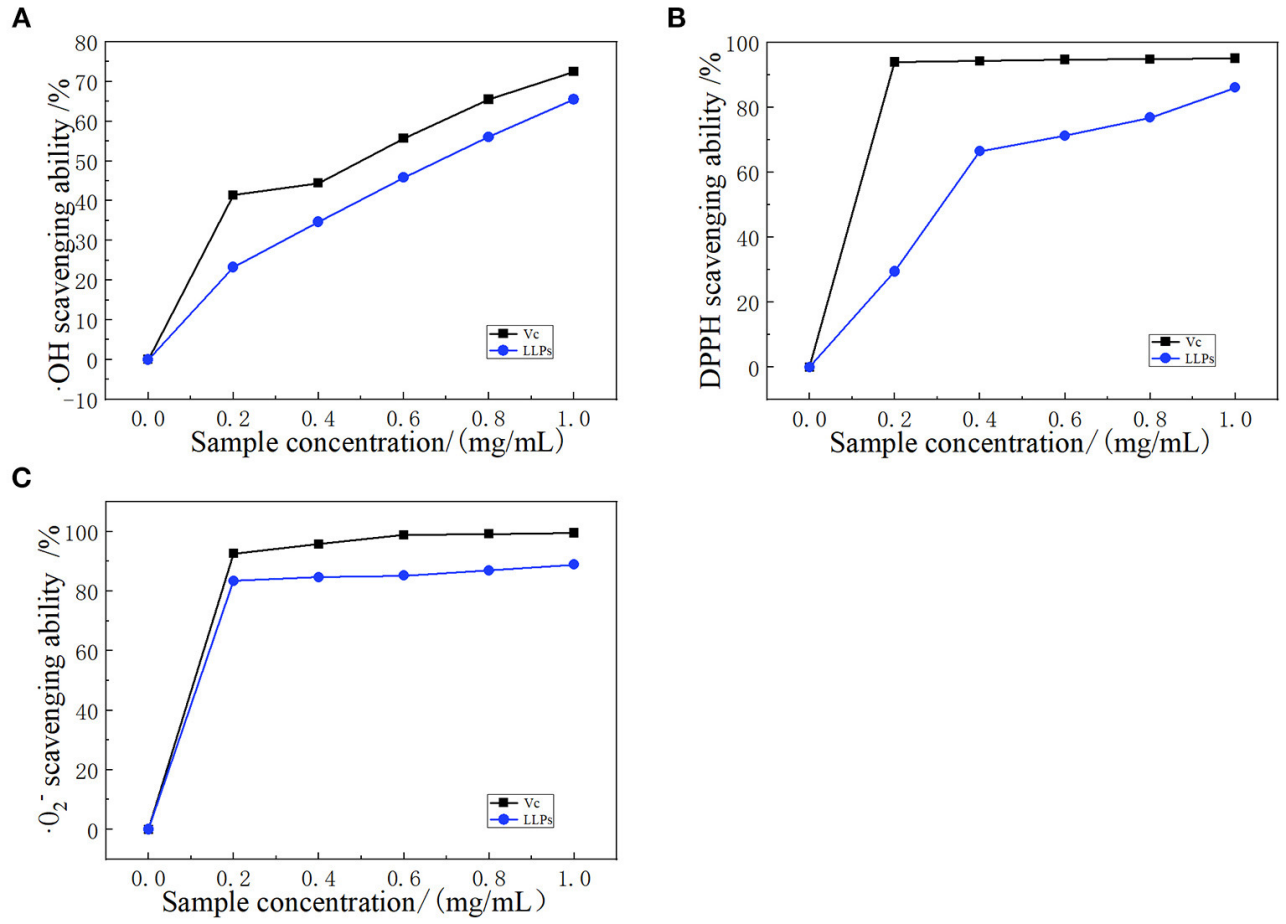
Scavenging effect of LLPs on **(A)** ·OH radicals compared with V_C_, **(B)** DPPH radicals compared with V_C_, and **(C)** ·${\text{O}}_{2}^{-}$ radicals compared with V_C_.

Hydroxyl radicals are a kind of compound harmful to organisms, and have a very high reactivity and can essentially attack and destroy living cells (33). Polysaccharides can be used as electron or hydrogen donors to remove hydroxyl radicals. Polysaccharides not only inhibit the production of ·OH, but also remove existing ·OH, indicating important antioxidant effects (34). The ·OH radical scavenging ability of LLPs was shown in Figure 6A, the scavenging ability of LLPs increased in the range of 0.2–1.0 mg/mL. Particularly, LLPs reached the highest values of 65.5% when the concentration was at 1.0 mg/mL, indicating that the scavenging ability of LLPs on ·OH free radical was concentration-dependent. At the same time, the semi-inhibitory concentration IC_50_ of LLPs was 0.63 mg/mL. The IC_50_ of V_C_ was 0.51 mg/mL. LLPs owe their scavenging ability of ·OH free radical to either the ·OH group possibly existing in their structure or the influence imposed by the density of electrons around heterocyclic carbon (35).

#### DPPH radical scavenging

Polysaccharides have many hydroxyl groups and most of them can donate hydrogen to reduce the DPPH radical (36). DPPH radical scavenging model is widely used in quantitative analysis of antioxidant ability. The Figure 6B showed the DPPH scavenging activity of LLPs increased from 23.3 to 65.5% with the concentration ranging from 0.0 to 1.0 mg/mL, indicating that the scavenging DPPH free radical of LLPs in a concentration-dependent manner (29). The concentration in the range of 0.0–0.4 mg/mL had a greater effect on DPPH radical scavenging ability, while the concentration had a smaller effect in the range of 0.4–1.0 mg/mL. LLPs at 1.0 mg/mL showed the highest DPPH radical scavenging ability of 86.1%. The scavenging ability of V_C_ at the same concentration was 95.1%. Moreover, the IC_50_ of LLPs was 0.32 mg/mL. Results showed that LLPs had a significant scavenging effect on DPPH free radicals, which was similar to results from a previous study (25).

#### Scavenging superoxide anion radicals

Superoxide radical can be found in the numerous biological and photochemical reactions. Although the superoxide radical is less reactive, it can reproduce other reactive oxygen species to cause the tissue damage and various diseases. As shown in Figure 6C, LLPs had significant scavenging activity on ·${\text{O}}_{2}^{-}$ radical. The scavenging value of LLPs reached 83.4% at 0.2 mg/mL, increased slowly in the range of 0.2–1.0 mg/mL. Particularly, the clearance rates of V_C_ and LLPs at 1.0 mg/mL were 99.5 and 88.9%, respectively. Although the superoxide radical scavenging activity of LLPs was a little weaker than those of V_C_, LLPs could be used as the superoxide radical inhibitors to reduce oxidative damage (37). In addition, the IC_50_ of LLPs was 0.30 mg/mL. Xu et al. reported that the scavenging activities of LLPs (LLP-1, LLP-2, LLP-3) were 93.20, 91.49, 96.83% when the concentration was 1.0 mg/mL (38).

## Conclusions

LLPs were extracted by ultrasonic assisted enzymatic method with polyethylene glycol. A LLPs yield of 14.75% was obtained at the optimized conditions: E/S ratio 1,400 U/g, pH 5.0, ultrasonic temperature 50 °C, ultrasonic time 30 min. The LLPs was mainly composed of galactose, glucose, rhamnose, and arabinose, with a ratio of 6.36: 76.03: 2.02: 7.09. LLPs were a α-d-glucan and pyranoid polysaccharide with characteristic absorption peaks of -OH, C=O, and C-H. The LLPs product had irregular distribution, dispersed structure and many holes. The LLPs showed a high ·OH radical scavenging activity, DPPH radical scavenging activity, and ·${\text{O}}_{2}^{-}$ radical scavenging activity of 62.1, 65.5, and 88.9%, with IC_50_ values of 0.63, 0.32, and 0.30 mg/mL, respectively. The results suggested that LLPs have potential application as natural antioxidant and food ingredients in functional food.

## Data availability statement

The raw data supporting the conclusions of this article will be made available by the authors, without undue reservation.

## Author contributions

The initiative for this work was from JY and DG designed experiments. DG and PG did experimental design of antioxidant. XY and HC prepared LLPs. XC and HX did the determination of purified LLPs structural. DG and HC wrote the manuscript. All authors read and approved the final manuscript.

## Funding

This work was supported by the Natural Science Foundation of China (No. 31960461); The Fundamental Research Funds for the Central Universities of Northwest Minzu University (31920220045 and BELTY201901); The Young Doctor Fund of Gansu Province (2021QB-147).

## Conflict of interest

Author HL was employed by Gansu Tobacco Industry Co. Ltd. The remaining authors declare that the research was conducted in the absence of any commercial or financial relationships that could be construed as a potential conflict of interest.

## Publisher's note

All claims expressed in this article are solely those of the authors and do not necessarily represent those of their affiliated organizations, or those of the publisher, the editors and the reviewers. Any product that may be evaluated in this article, or claim that may be made by its manufacturer, is not guaranteed or endorsed by the publisher.
